# Omega-3 dietary supplementation combined with exercise to keep telomere integrity in the liver of aged obese female mice

**DOI:** 10.1007/s13105-026-01139-5

**Published:** 2026-01-29

**Authors:** Paola Elizabeth Gámez-Macías, Elisa Félix-Soriano, Neira Sáinz, Amelia Martí del Moral, Sonia García-Calzón, María Jesús Moreno-Aliaga, Pedro González-Muniesa

**Affiliations:** 1 Fac Pharm & Nutr, Dept Nutr Food Sci & Physiol, University of Navarra, Irunlarrea 1, Pamplona, 31008 Spain; 2 Center for Nutrition Research, University of Navarra, Pamplona, Spain; 3 Centro de Investigación Biomédica en Red de Fisiopatología de la Obesidad y Nutrición (CIBEROBN), Instituto de Salud Carlos III (ISCIII), Madrid, Spain; 4 Navarra Institute for Health Research (IdiSNA), Pamplona, Spain

**Keywords:** Overweight, PUFAs, Inflammation, MASLD, Physical activity

## Abstract

Telomere shortening is a key marker of cellular aging and linked to pathologies such as liver disease. Oxidative stress and inflammation (hallmarks of obesity) contribute to telomere shortening, while omega-3 (DHA) and exercise may counteract these effects by enhancing cellular homeostasis. This study aims to analyze the influence of DHA supplementation and/or exercise over one year on liver telomere length in obese aged mice. Two-month-old female mice were fed either a control or high-fat diet (HFD) for four months. Diet-induced obese (DIO) mice were then assigned to one of four groups: (1) DIO, maintained on an HFD; (2) DIO + EX, subjected to exercise; (3) DIO + DHA, fed an HFD supplemented with DHA; and (4) DIO + DHA + EX, subjected to both exercise and DHA supplementation. The intervention continued until the mice reached 18 months of age. The DIO group showed significant telomere attrition, which was prevented only when omega-3 and exercise were combined. Additionally, only the combined DHA and exercise group improved the expression of genes related to oxidative stress (*Sirt3*,* Foxo3*,* Sod1*,* Cat*). Interestingly, DHA and exercise separately reduced pro-inflammatory cytokine Il-1b expression compared to the control group, but not when combined. These results indicate that DHA combined with physical exercise could be an effective strategy to maintain telomere integrity in aged obese female mice, due to their antioxidant properties.

**Supplementary Information:**

The online version contains supplementary material available at 10.1007/s13105-026-01139-5.

## Key points

Telomere attrition contributes to liver disease progression.

Omega-3 and exercise could slow telomere shortening in obese aged mice.

DHA supplementation and exercise may stimulate Sod1 and Cat expression.

**Supplementary Information:**

The online version contains supplementary material available at 10.1007/s13105-026-01139-5.

## Introduction

Telomere attrition is one of the hallmarks of cellular senescence [1] and has also been implicated in age-related diseases such as atherosclerosis, osteoporosis, type 2 diabetes, liver disease, etc [2]. Telomeres are located at the end of chromosomes and contain repeated sequences of TTAGGG that protect and regulate the biological function of DNA [2]. During every replication cycle, telomeres shorten until the cells reach a replicative senescence state called the “Hayflick limit”, at which time they cease to divide [1]. An overproduction of reactive oxygen species (ROS) accelerates telomere shortening which contributes to the aging process [3] as well as the progression of certain illnesses, such as liver diseases [2, 4, 5]. Inflammation and mitochondrial dysfunction are two factors observed in obesity [6, 7] and aging [8, 9] that significantly increase ROS production (oxidative stress) [8].

Lifestyle interventions such as exercise and nutritional supplementation have emerged as effective strategies to mitigate oxidative stress and inflammation, which are key drivers of cellular aging and metabolic disease. Exercise is known to enhance endogenous antioxidant defenses and reduce pro-inflammatory cytokines, thereby contributing to telomere maintenance and cellular health [10]. Similarly, omega-3 polyunsaturated fatty acids, particularly docosahexaenoic acid (DHA), have been shown to increase antioxidant enzyme production (e.g., superoxide dismutase and catalase) and attenuate telomere attrition by lowering oxidative stress levels [11].

Polyunsaturated fatty acids (PUFAs) have antioxidant and anti-inflammatory properties [12]. DHA, an omega-3 PUFA, increases the production of antioxidant enzymes such as superoxide dismutase (SOD) and catalase enzyme (CAT), keeping ROS levels under control [13–15]. Additionally, DHA inhibits the activation of the nuclear factor-κB (NF-κB) and, therefore, ameliorates liver inflammation [16, 17].

It is well known that exercise confers multiple health benefits. However, the magnitude and nature of these effects appear to depend on the type and intensity of the exercise [18]. Evidence indicates that moderate exercise training attenuates oxidative damage by upregulating the expression of antioxidant enzyme genes, such as SOD and CAT [18]. Furthermore, moderate exercise has been shown to reduce the release of pro-inflammatory cytokines, including tumor necrosis factor-alpha (TNF-α) and interleukin-1 beta (IL-1β) [19]. In contrast, increased levels of these proteins have been reported in the livers of mice subjected to intense exercise [20].

However, despite the positive impact of these two strategies on oxidative stress and inflammation, it is unclear whether they contribute to maintaining liver telomere integrity. The OBELEX research project evaluated the effect of omega-3 and exercise on aged obese female mice. The findings of this project have been published and showed that long-term DHA supplementation and/or exercise training can ameliorate liver steatosis in 18-month-old DIO female mice [21]. In the present article, these samples from the OBELEX project were used to determine if long-term supplementation and/or exercise could contribute to keep liver telomere integrity. This therapy could potentially help as a novel treatment to prevent and/or slow down steatosis progression by mitigating telomere attrition.

## Materials and methods

### Animal experimental design

This study was conducted with samples obtained from the OBELEX project [21, 22]. Briefly, female C57BL/6J mice (seven weeks old) were purchased from Harlan Laboratories (Barcelona, Spain). Animals were housed at the animal facilities of the University of Navarra under strictly controlled conditions (22 ± 2 °C, with a 12 h light–dark cycle, relative humidity, 55 ± 10%). The Ethics Committee for Animal Experimentation of the University of Navarra approved all experiments (Protocol 113–15), performed following EU Directive 2010/63/EU (https://www.unav.edu/investigacion/nuestra-investigacion/etica-para-la-investigacion#ceea). The number of animals was estimated considering factors such as significance level (5%), and statistical power (90%) to determine the appropriate sample size. After acclimation, mice were divided into two experimental groups: (i) Control group (*n* = 10) fed a normal control diet (20% proteins, 67% carbohydrates, and 13% lipids, provided by Harlan Teklad Global Diets, Harlan Laboratories, Indianapolis, IN, USA); and (ii) Diet-induced obese (DIO) group fed a high fat saturated diet (HFD: 20% proteins, 35% carbohydrates, and 45% lipids, provided by Research Diets, Inc., New Brunswick, NJ, USA). For four months, mice were fed these two diets *ad libitum*. Then, the DIO group was divided into 4 experimental groups: (i) DIO group (*n* = 10) that continued with the HFD; (ii) DIO + EX group (*n* = 10) fed with the same HFD than the DIO group in combination with treadmill exercise training; (iii) DIO + DHA group (*n* = 10) fed with the HFD containing a DHA-rich n-3 PUFA concentrate from fish oil replacing 15% of dietary lipids (Research Diets Inc., New Brunswick, NJ, USA); and (iv) DIO + DHA + EX group (*n* = 10) fed with the HFD containing the DHA-rich n-3 PUFA concentrate, in combination with exercise training.

The DHA-rich n-3 PUFA concentrate provided by Solutex, Spain (SOLUTEX0063TG), contained 683.4 mg DHA/g and 46.7 mg EPA/g, with a total n-3 PUFA content of 838.9 mg/g in the triglyceride form. Since the DHA-rich n-3 PUFA concentrate included mixed tocopherols (2 mg/g of Covi-ox^®^ T-79EU), the high-fat diets (HFD) of the DIO and DIO + EX groups were supplemented with the same tocopherol dose to ensure consistency across treatments. Further details of the diets can be found in Supplementary Table S1.

For each group, cages (5 animals per group) were randomly assigned at baseline (2 months of age) and after obesity induction (6 months of age). To avoid mistakes, the tails of the animals were marked with different colored markers to identify different groups. Furthermore, all the measurements and treatments were always approached following controlled but changing order to avoid potential confounders.

The experiments and outcomes assessment were not blinded. Animals were subjected to these experimental conditions until the age of 18 months was reached. At the end of the experimental period, mice were sacrificed after overnight fasting, and samples were collected and kept at − 80º C for further analysis. Prior to sacrifice, body composition was assessed using nuclear resonance technology (EchoMRI-100–700; Echo Medical Systems, Houston, TX, USA), as previously described [23]. It is important to note that one mouse was excluded from the control group because it developed obesity. Furthermore, some mice died before the endpoint: DIO + EX: 1; DIO + DHA: 4; and DIO + DHA + EX: 1. Weight loss exceeding 20%, immobility, cachexia, or an unresponsive response to stimulation were considered humane endpoints for the study.

### Training protocol

The DIO + EX and DIO + DHA + EX groups were subjected to a treadmill exercise program (LE8710M, Panlab, Barcelona, Spain) from 6 until 18 months old. Initially, the mice were allowed to adapt to the treadmill by running for 10 min on two consecutive days on a treadmill (the first day at a speed of 3 m/min and the second day at a speed of 4.8 m/min). Afterward, the mice were trained at low intensities from 6 to 10 months of age (3 m/min for 5 min, increasing to 4.8 m/min for 5 min, then reaching a maximum of 7.2 m/min for 20 min at 0% slope) during 3 alternate days per week. At 10 months of age, the number of sessions and the speed of the training were increased to 5 days per week during 5 weeks with the following protocol: the initial running speed and time were 5 m/min for 5 min, then increased to 8 m/min for 5 min, then increased to 12 m/min for 20 min at 0% slope. In the following seven months, the exercise protocol was maintained, but the number of sessions was reduced from five to three per week. For the same duration as the exercise group, the mice in the non-exercise groups were left on the treadmill without running. Mice were sacrificed 30 h after the last training session, following an overnight fasting period.

### Telomere length assessment

Genomic DNA was extracted from the liver using the DNeasy Blood & Tissue Kit (Qiagen) according to the manufacturer’s protocol at 18 months old. A Monochrome Multiplex Real-time Quantitative PCR (MMQPCR) method was used to analyze telomere based on Cawthon’s guidelines [24] and our previous expertise in telomere length assessment [25]. Supplementary Table S2 lists the primer sequences for qPCR of telomere and albumin. A calibration curve based on a reference DNA sample was included in the plate and all samples were run in triplicate for quality control. Telomere (T) length was expressed as a T/S ratio, where S (simple copy gene) was albumin.

### Hepatic gene expression analysis (qRT-PCR)

Isolation of RNA from liver tissue at 18 months old was carried out using TRIzol^®^ Reagent (Invitrogen, ThermoFisher Scientific, Waltham, MA, USA). In the next step, RNA (1–5 g) was incubated with DNase I (RapidOut DNA Removal kit, Thermo Fisher Scientific, Waltham, MA, USA) at 37 °C for 30 min. The quality and concentration of RNA were analyzed using the Nanodrop Spectrophotometer ND1000 (Nanodrop Technologies, Inc. Wilmington, NC, USA). Then, retrotranscription to cDNA was performed using High-Capacity cDNA Reverse Transcription (Applied Biosystems, Foster City, CA) according to the manufacturer’s instructions. As a final step, a quantitative PCR was carried out using the Touch Real-Time PCR System (C1000 + CFX384, BIO-RAD, Hercules, CA, USA). Gene expression was assessed using Power SYBR Green PCR Master Mix. Detailed primer sequences for qPCR of *Sirt3*,* Foxo3*,* Sod1*,* Cat*,* Il-1β*, and *Il-10* can be found in Supplementary Table S3. Relative gene expression was determined by the 2 − ^ΔΔCT^ method, and the expression data were normalized with the *36b4* gene expression. For quality control, each sample was run in duplicate, and the mean values were analyzed statistically.

### Serum transaminases and liver triglycerides measurement

The serum levels of alanine amino transferase (ALT) and aspartate amino transferase (AST) were determined following a 12-hour fasting period using a Pentra C200 autoanalyzer (Roche Diagnostic, Basel, Switzerland). Liver triglycerides from the OBELEX project were measured as previously reported by Yang et al. [21]. These previously reported data were used for correlation analysis.

### Statistical analysis

The one-way ANOVA test was conducted when the data were normally distributed. Normality was screened using the Kolmogorov-Smirnov and Shapiro–Wilk tests. When the data were not normal, Kruskal-Wallis’s test was used. After a post-hoc Tukey or Tamhane test (if heteroscedasticity exists) was conducted to assess differences between groups. To identify interactions between factors (omega 3 and exercise), a two-way ANOVA test was performed.

In addition, spearman rank correlation analysis was conducted. A p-value < 0.05 was considered statistically significant. Data analysis was conducted using GraphPad Prism 10 for Mac version 10.0.2.

## Results

### Effect of exercise and omega-3 supplementation on body weight and composition

**Fig. 1:**
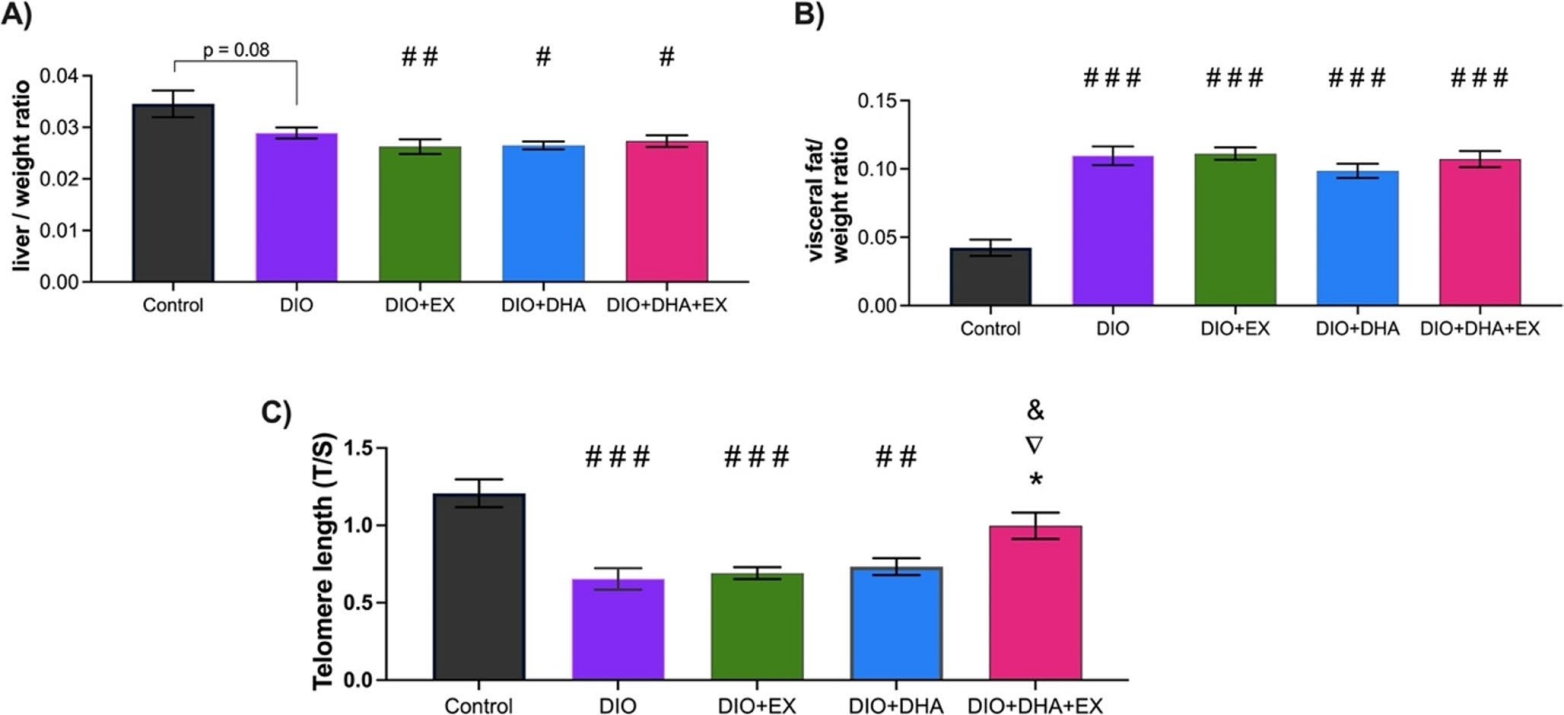
Analysis at 18 months of age of the effects of exercise and/or omega-3 supplementation on: (**A**) liver/body weight ratio, and (**B**) visceral fat/body weight ratio. (**C**) Telomere length, measured in the liver of mice at 18 months, is expressed as the T/S ratio (telomere repeat copy number/single gene copy number). Data are presented as mean ± SEM, with *n* = 6–8 per group. * *p* < 0.05 vs. DIO; #*p* < 0.05, # # *p* < 0.01, # # # *p* < 0.001 vs. control; ▽ *p* < 0.05 vs. DIO + EX; & *p* < 0.05 vs. DIO + DHA

In order to assess the effects of treatment on body weight and composition, two ratios were calculated. As can be seen in Fig. 1A, the liver/weight ratio was lower in all groups fed a high-fat diet compared with the control group, but no differences were found between mice subjected to treatments. Opposite to this result, the visceral fat/weight ratio was higher in all DIO groups than the control group and the treatments did not show any effect (Fig. 1B).

### Effect of exercise and omega-3 supplementation on telomere length

The results showed that mice fed a high-fat diet (DIO group) had shorter telomeres than the control group reducing their telomeres by 43.8% (SEM ± 7.5). Even when obese mice were fed a long-term dietary supplementation with omega-3 (DIO + DHA group) or exercised (DIO + EX group), they were not able to counteract the effect of HFD on telomere shortening, showing a reduction of by 40.1% (SEM ± 5.1) and 39.9% (SEM ± 9.2), respectively. Nevertheless, when omega-3 supplementation and exercise were combined (DIO + DHA + EX group), the telomere length was significantly less shortened than in the DIO (*p* = 0.01), the DIO + EX (*p* = 0.04) and the DIO + DHA (*p* = 0.03) groups after 1 year of treatment (Fig. 1C). In addition, the two-way ANOVA to evaluate the interaction between exercise and omega-3 supplementation on telomere length resulted significative (*p* < 0.001).

### Effect of exercise and omega-3 supplementation on gene expression related to oxidative stress homeostasis in the liver

**Fig. 2:**
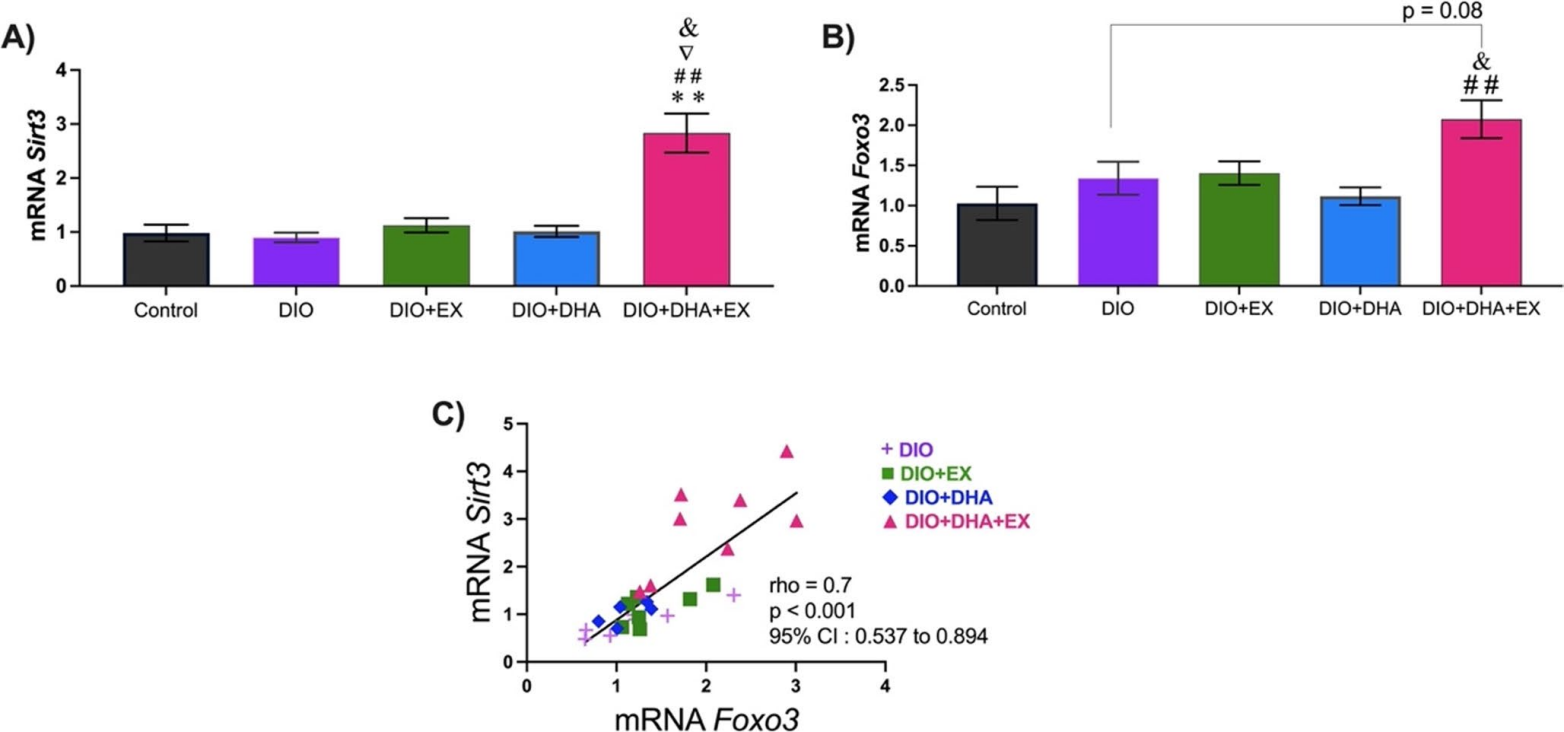
Effect of long-term dietary supplementation with omega-3-rich DHA and treadmill exercise, either alone or in combination on: (**A**) gene expression of *Sirt3*, and (**B**) gene expression of *Foxo3* in the liver at 18 months old. (**C**) Spearman rank correlation between *Sirt3* and *Foxo3* (*n* = 31). Data are presented as mean ± SEM, with *n* = 6–10 per group. **p < 0.01 vs. DIO; # *p* < 0.05 vs. control; ▽ *p* < 0.05 vs. DIO + EX; & *p* < 0.05 vs. DIO + DHA

The effect of DHA and exercise on sirtuin 3 (Sirt3), forkhead box O3 (Foxo3), and the antioxidant genes superoxide dismutase 1 (Sod1) and catalase (Cat) was analyzed. As shown in Fig. 2A, only the combined DHA and exercise treatment significantly increased *Sirt3* expression (*p* = 0.009 vs. control; *p* = 0.008 vs. DIO; *p* = 0.01 vs. DIO + EX; *p* = 0.01 vs. DIO + DHA). Additionally, the DIO + DHA + EX group showed higher *Foxo3* expression compared to the control (*p* = 0.009) and DIO + DHA (*p* = 0.04) groups (Fig. 2B). A positive correlation was observed between *Sirt3* and *Foxo3* expression (Fig. 2C). Furthermore, a significant interaction between exercise and omega-3 supplementation was found for *Sirt3* and *Foxo3* expression (*p* < 0.001 and *p* = 0.04, respectively).

**Fig. 3:**
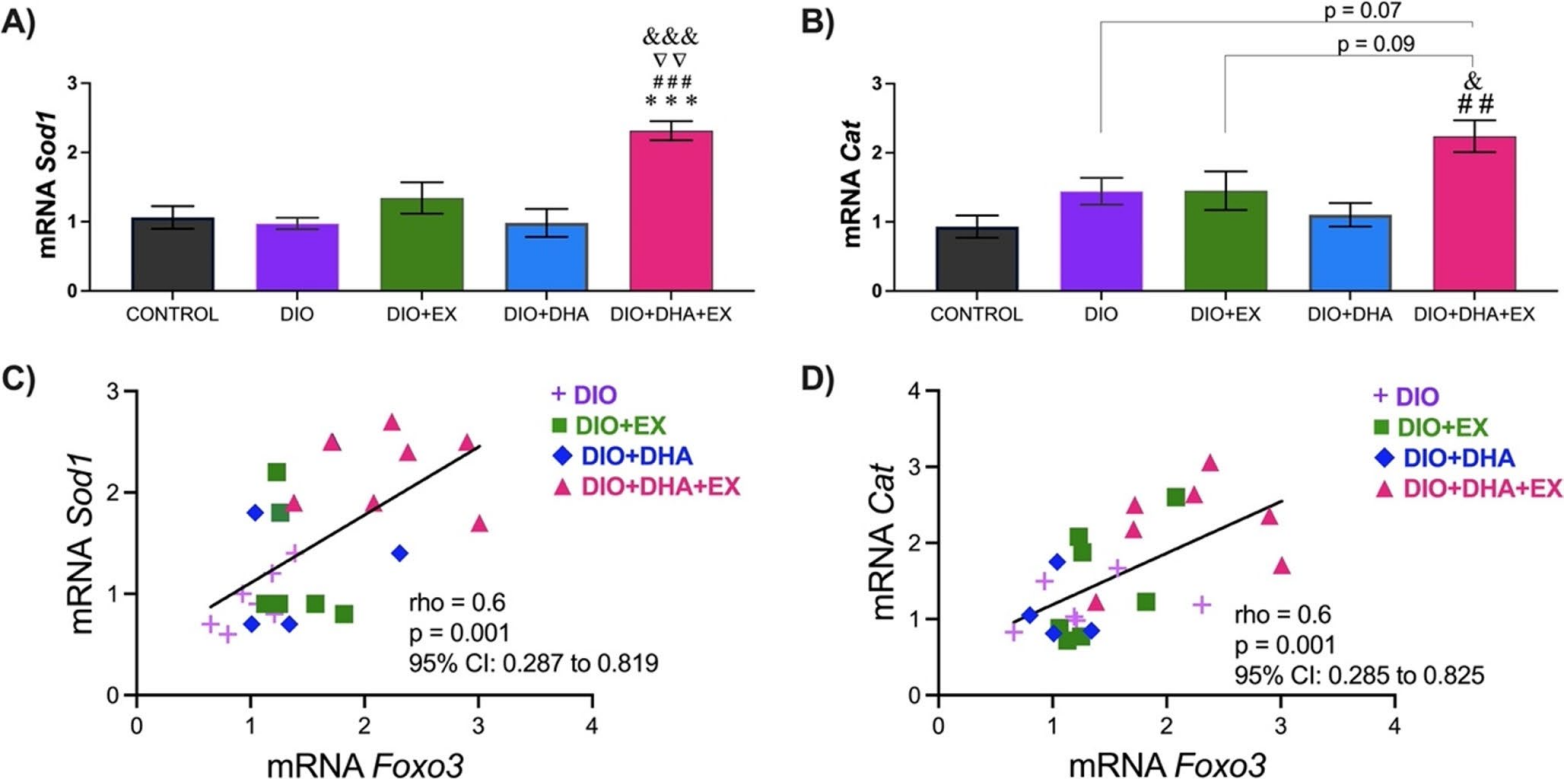
Comparison of the effect of omega-3-rich DHA supplementation and/or exercise on the expression of antioxidant genes in the liver at 18 months of age: (**A**) *Sod1* gene expression, (**B**) *Cat* gene expression. Data are presented as mean ± SEM (*n* = 6–10 per group). ***p < 0.001 vs. DIO; # # *p* < 0.01, # # # *p* < 0.001 vs. Control; ▽▽ *p* < 0.01 vs. DIO + EX; & *p* < 0.05, &&& *p* < 0.001 vs. DIO + DHA. (**C**) Spearman rank correlation between *Sod1* and *Foxo3* (*n* = 30). (**D**) Spearman rank correlation between *Cat* and *Foxo3* (*n* = 30)

Results of the present study showed that obese mice receiving DHA supplements along with exercise (DIO + DHA + EX group) exhibited increased expression of the *Sod1* gene compared to the rest of the experimental groups (*p* < 0.001 vs. control; *p* < 0.001 vs. DIO; *p* = 0.002 vs. DIO + EX; *p* < 0.001 vs. DIO + DHA, see Fig. 3A). Similarly, as shown in Fig. 3B, the DIO + DHA + EX group showed higher *Cat* expression compared to the control (*p* = 0.001) and DIO + DHA (*p* = 0.01) groups. Additionally, the interaction between DHA supplementation and exercise was found to be significant for both genes (*p* = 0.007 for *Sod1* and *p* = 0.02 for *Cat*). Moreover, a positive correlation was observed between these antioxidant genes and *Foxo3* (see Fig. 3C, D).

### Effect of exercise and omega-3 supplementation on the expression of inflammation-related genes in the liver

**Fig. 4:**
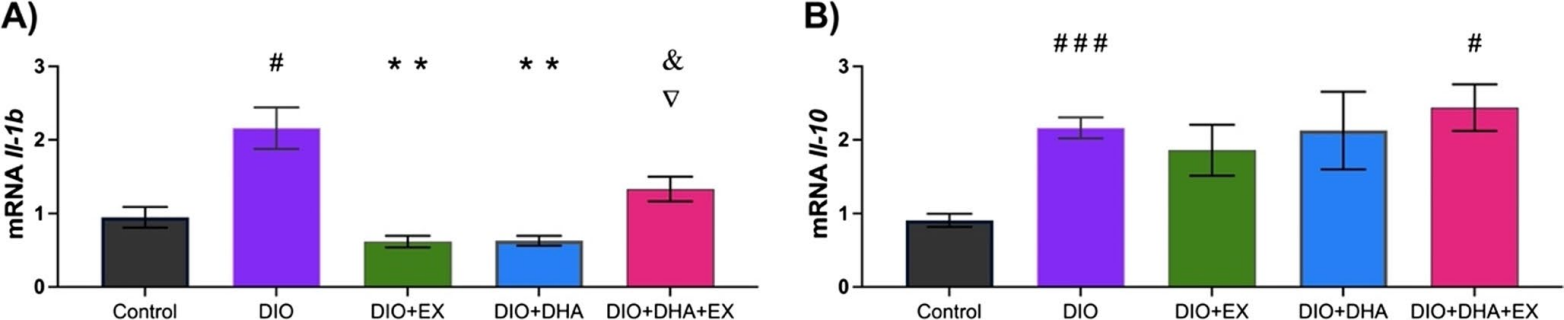
Effects of long-term dietary supplementation with omega-3-rich DHA, alone or combined with exercise, on liver inflammation genes at 18 months. (**A**) *Il-1b* expression. (**B**) *Il-10* expression. Data are presented as mean ± SEM (*n* = 6–10). **p < 0.001 vs. DIO; #*p* < 0.05, # # #*p* < 0.001 vs. Control; ▽*p* < 0.05 vs. DIO + EX; &*p* < 0.05 vs. DIO + DHA

Lastly, it was analyzed whether the treatments could ameliorate liver inflammation in mice by quantifying the gene expression of two cytokines: the pro-inflammatory interleukin-1β (*Il-1b*) and the anti-inflammatory interleukin-10 (*Il-10)*. It was found that the DIO mice showed higher levels of *Il-1b* than the control group (*p* = 0.02). Notably, mice treated with omega-3 (DIO + DHA), or exercise (DIO + EX) showed a reduced expression of this cytokine (*p* = 0.005 and *p* = 0.006, respectively) compared to the DIO group. However, this effect was not observed when the treatments were combined (Fig. 4A).

Regarding the anti-inflammatory *Il-10* gene expression, the DIO group exhibited higher levels of *Il-10* expression than the control group (*p* < 0.001). Similarly, the combination of the treatments did not reverse this effect (control vs. DIO + DHA + EX group, *p* = 0.01; Fig. 4B).

### Telomere length correlations with body composition, transaminases, and gene expression

In addition, correlation analyses were performed to assess the relationship between telomere length, weight, and body composition; however, the results were not significant. Moreover, correlation analyses were conducted to evaluate the link between telomere length and serum transaminases (ALT and AST). A negative correlation was observed between ALT and telomere length, whereas no significant correlation was found with AST. Additionally, the correlation between telomere length and the expression of genes related to oxidative stress was evaluated. The results showed a positive correlation between telomere length and the expression of *Sirt3*, as well as the *Sod1* gene. Finally, no significant correlations were found between telomere length and inflammation-related genes (Table 1).

**Table 1:** Spearman rank correlation between telomere length and body composition, serum transaminases and gene expression in the liver at 18 months old (*n* = 25–29)

Body composition	*r*	95% CI	*p* value
Weight	−0.16	−0.22–0.50	ns
Fat mass	0.08	−0.29–0.45	ns
Liver weight	0.14	−0.25–0.50	ns
Transaminase			
ALT	−0.41	−0.70–0.001	0.04
AST	−0.26	−0.63–0.19	ns
Gene			
*Sirt3*	0.45	0.07–0.71	0.01
*Foxo3*	0.26	−0.13–0.59	ns
*Sod1*	0.49	0.12–0.74	0.001
*Cat*	0.36	−0.04–0.67	0.07
*Il-1b*	0.02	−0.35–0.40	ns
*Il-10*	−0.02	−0.43–0.39	ns

*ns* no significative

## Discussion

The present study examined whether long-term DHA consumption and/or regular exercise could help to maintain telomere integrity in the liver of aged DIO female mice from the OBELEX project. Our findings suggest that the combination of DHA supplementation and exercise exerts a synergistic effect on hepatic antioxidant defenses, which may explain the attenuated telomere shortening observed in this group. Specifically, SOD1 and CAT expression increased significantly only under the combined intervention, whereas anti-inflammatory markers improved with either treatment alone. This pattern indicates that telomere protection is more closely associated with enhanced ROS detoxification than with additional reductions in inflammation. Mechanistically, DHA reduces oxidative damage by integrating into cellular membranes, decreasing lipid peroxidation [26], improving mitochondrial efficiency [27], while exercise induces adaptive upregulation of endogenous antioxidant enzymes [28]. Together, these complementary mechanisms amplify antioxidant capacity beyond the effect of either intervention alone. This interpretation aligns with previous evidence showing that omega-3 PUFAs influence telomere biology by reducing oxidative stress [11] and that omega-3 supplementation combined with exercise produces synergistic antioxidant effects in animal models [29].

Moreover, in the present study the joined effect of DHA and exercise that upregulated gene related to oxidative stress (*Sirtuin3*,* Foxo3*,* Sod1*, and *Cat)*, which could have led to the decrease in ROS levels. In addition, a negative correlation between ALT and telomere length, and also with *Sirt3* was found.

Previous studies conducted in male mice under non-obesogenic conditions provide relevant context. One study [30] reported that DHA supplementation increased SOD levels in male mice fed a standard chow diet. Similarly, Wu et al. [31] found that high-intensity acute exercise increased *Sod1* and *Cat* expression in male mice that were not consuming a high-fat diet. These findings suggest that factors such as sex, exercise intensity, and obesity may influence the responsiveness of antioxidant pathways, indicating that moderate exercise or DHA supplementation alone might be insufficient to enhance antioxidant gene expression in obese female mice.

Interestingly, the pro-inflammatory cytokine *Il-1β* associated with an increased risk of MASLD [32], was downregulated in mice with either DHA consumption or exercise treatments as it was observed in other studies [33, 34] but the combination of both did not show any improvement. Moreover, the present study showed that exercise alone did not ameliorate the adverse effect of a high-fat diet on telomeres length, despite its effect on the reduction of inflammatory cytokine *Il-1β* gene expression. In contrast, the study of Ludlow et al. [35] in male and female middle-aged mice (12 months old) following a standard diet reported that those with exercise had longer liver telomeres than those without. These findings suggest that exercise may not be sufficient to counteract telomere shortening caused by a long-term high-fat diet in old female mice (18 months old).

In addition, our results showed that DHA consumption alone did not have a positive effect on liver telomere length of obese mice, similar to the findings by Wu, et al. [36]. In contrast, a recent study in male mice reported that DHA can prevent telomere shortening in liver tissue [37]. In that study, however, in addition to the sex difference, the telomere length analysis was conducted on middle-aged mice (12 months of age), whereas in the current study, the analysis was conducted in old mice (18 months of age), and their dietary intervention was initiated at 2 months of age, whereas in the present study, DHA consumption was initiated at 6 months of age. Hence, implementing prevention strategies at an early age may be more beneficial.

The anti-inflammatory cytokine, IL-10, inhibits monocyte differentiation, thus suppressing inflammatory cytokine release. Moreover, IL-10 delays liver regeneration, by reducing extracellular matrix protein formation and lowering inflammation, slowing fibrosis [38]. Surprisingly, *Il-10* gene expression increased in both the DIO and DIO + DHA + EX groups, possibly as a result of an increase in the proinflammatory cytokine *Il-1b*. This result is similar to Guerra et al. [39] who conducted a study in female mice fed a high-fat diet for 6 months and observed an increase in expression of *Il10* cytokine in contrast to mice fed a standard diet.

The main limitation of the present study is that only female mice were used, but there were several scientific reasons for this experimental design. For example, numerous previous studies have examined telomere attrition and liver oxidative stress, as well as n-3 PUFA supplementation and exercise, but most of them have focused exclusively on male mice [30, 31, 36]. This was one of the reasons for selecting females in the present study. In addition, female mice have longer telomeres than males, which may partly account for sex differences in aging [40]. It has also been reported that estrogen deficiency in mice fed a high-fat diet accelerates the progression of nonalcoholic steatohepatitis [41]. Moreover, estrogen exerts protective effects on mitochondria, and reductions in this hormone could therefore contribute to increased oxidative stress [42]. However, hormonal levels were not directly measured in the present study. By 18 months of age, female mice are acyclic, and exhibit reduced circulating estrogen and progesterone [43]. These age-related hormonal changes may modulate oxidative stress and influence telomere attrition.

In summary, a combination of long-term DHA consumption and moderate exercise appears to slow down telomere shortening through mechanisms related to the regulation of oxidative stress in the liver of aged obese mice. It is important to investigate the mechanisms by which these treatments together achieve an effect while following only one of the strategies does not seem to be enough to prevent telomere shortening.

## Supplementary Information

Below is the link to the electronic supplementary material.

ESM 1 Supplementary tables (DOCX 23.3 KB)

## Data Availability

No datasets were generated or analysed during the current study.
