# Supplementary material for: Omega-3 dietary supplementation combined with exercise to keep telomere integrity in the liver of aged obese female mice
Source: J Physiol Biochem. 2026 Jan 29;82(1):5. doi: 10.1007/s13105-026-01139-5 (PMC12855355; doi:10.1007/s13105-026-01139-5)
Supplement: Supplementary file 1 — Supplementary tables (DOCX 23.3 KB) [file 13105_2026_1139_MOESM1_ESM.docx]

**Supplementary Table S1.** Detailed description of the three diets that were used during the study

| **Diet** | **Standard HFD** | | |  | **HFD Tocopherols*** | |  | | **HFD + DHA-rich *n-*3  PUFA Marine Oil**** | |
| --- | --- | --- | --- | --- | --- | --- | --- | --- | --- | --- |
| **Product #** | **D12451** | | |  | **D16112302** | |  | | **D16112301** | |
| **Macronutrient %** | **g** | | **kcal** |  | **g** | **Kcal** |  | **g** | | **Kcal** |
| Protein | 23.7 | | 20 |  | 23.7 | 20 |  | 23.7 | | 20 |
| Fat | 23.6 | | 45 |  | 23.6 | 45 |  | 23.6 | | 45 |
| Carbohydrate | 41.4 | | 35 |  | 41.4 | 35 |  | 41.4 | | 35 |
| Total |  | | 100 |  |  | 100 |  |  | | 100 |
| kcal/g | 4.73 | |  |  | 4.73 |  |  | 4.73 | |  |
| **Ingredient** |  | |  |  |  |  |  |  | |  |
| Casein | 200 | | 800 |  | 200 | 800 |  | 200 | | 800 |
| L-Cystine | 3 | | 12 |  | 3 | 12 |  | 3 | | 12 |
| Soybean Oil | 25 | | 225 |  | 25 | 225 |  | 25 | | 225 |
| Lard | 177.5 | | 1598 |  | 177.5 | 1598 |  | 147.1 | | 1324 |
| High DHA Marine Oil (Solutex)* | | 0 | 0 |  | 0 | 0 |  | 30.4 | | 274 |
| Corn Starch | 72.8 | | 291 |  | 72.8 | 291 |  | 72.8 | | 291 |
| Maltodextrin 10 | 100 | | 400 |  | 100 | 400 |  | 100 | | 400 |
| Sucrose | 172.8 | | 691 |  | 172.8 | 691 |  | 172.8 | | 691 |
| Cellulose | 50 | | 0 |  | 50 | 0 |  | 50 | | 0 |
| Vitamin Mix V10001 | 10 | | 40 |  | 10 | 40 |  | 10 | | 40 |
| Choline Bitartrate | 2 | | 0 |  | 2 | 0 |  | 2 | | 0 |
| Mixed tocopherols | 0 | | 0 |  | 0.061 | 0 |  | 0 | | 0 |
| FD&C Yellow Dye # 5 | 0 | | 0 |  | 0 | 0 |  | 0.05 | | 0 |
| FD&C Red Dye # 40 | 0.05 | | 0 |  | 0 | 0 |  | 0 | | 0 |
| FD&C Blue Dye # 1 | 0 | | 0 |  | 0.05 | 0 |  | 0 | | 0 |
| TOTAL | 858.15 | | 4057 |  | 858.21 | 4057 |  | 858.15 | | 4057 |
| DHA content in the diet (g/kg) | 0 | | 0 |  | 0 | 0 |  | 24.2 | | 0 |
| EPA content in the diet (g/kg) | 0 | | 0 |  | 0 | 0 |  | 1.65 | | 0 |

* The DHA-rich *n-*3 PUFA concentrate contains 2 mg/g of mixed tocopherols to prevent oxidation.

**A standard high-fat diet (HFD) including the same quantity of tocopherols contained in the HFD formulated with the DHA-rich *n-*3 PUFA concentrate (SOLUTEX), containing 683.4 mg DHA/g and 46.7 mg EPA/g, as triglycerides. Diets were formulated and prepared by Research Diets Inc

| **Suplemmentary Table S2**. Sequence used for measuring telomeres and albumin. | |
| --- | --- |
| **Oligo name** | **Sequence 5' - 3'** |
| *Telg* | 5' - ACACTAAGGTTTGGGTTTGGGTTTGGGTTTGGGTTAGTGT - 3' |
| *Telc* | 5' - TGTTAGGTATCCCTATCCCTATCCCTATCCCTATCCCTAACA - 3' |
| *ALBs* | 5' - CGGCGGCGGGCGGCGCGGGCTGGGCGGAAATGCTGCACAGAATCCTTG - 3' |
| *ALBa* | 5' - GCCCGGCCCGCCGCGCCCGTCCCGCCGGAAAAGCATGGTCGCCTGTT -3' |

| **Suplemmentary Table S3**. Primer sequences used for qPCR. | | |
| --- | --- | --- |
| **Gen** | **Forward** | **Reverse** |
| *Sirt3* | GTCCACCAGCCTTTCCACAC | CTGACTTCGCTTTGGCAGAT |
| *Foxo3* | TACGAGTGGATGGTGCGCTGT | TCATTCTGAACGCGCATGAAGC |
| *Cat* | GGAGGCGGGAACCCAATAG | GTGTGCCATCTCGTCAGTGAA |
| *Sod1* | GAGACCTGGGCAATGTGACT | GTTTACTGCGCAATCCCAAT |
| *Il-1𝛽* | GCCACCTTTTGACAGTGATGAG | GACAGCCCAGGTCAAAGGTT |
| *Il-10* | AAGGCAGTGGAGCAGGTGAA | CCAGCAGACTCAATACACAC |
| *36b4* | CACTGGTCTAGGACCCGAGAAG | GGTGCCTCTGGAGATTTTCG |
